# Sutureless socket technique after removal of third molars: a multicentric, open, randomized controlled trial

**DOI:** 10.1186/s12903-022-02287-y

**Published:** 2022-06-26

**Authors:** Sarah Takadoum, Grégory Douilly, Marie de Boutray, Sarah Kabani, Eric Maladière, Christophe Demattei, Philippe Lapeyrie

**Affiliations:** 1 Service de Chirurgie Orale, Department of Oral Surgery, CHU Nîmes, CHRU de Nîmes - Hôpital Universitaire Carémeau, University of Montpellier, Place du Professeur Debré, 30029 Nîmes Cedex 9, France; 2 Pôle de Chirurgie Orale, 320 rue René Cassin, 84000 Avignon, France; 3 Department of Maxillofacial Surgery, Montpellier Regional University Hospital – Gui de Chauliac Hospital, Montpellier, France; 4 Department of Biostatistics, Clinical Epidemiology, Public Health and Innovation in Methodology (BESPIM), CHU Nîmes, University of Montpellier, Nîmes, France; 5 Department of Maxillofacial Surgery, Perpignan Hospital Perpignan, Perpignan, France

**Keywords:** Molar, third, Tooth, impacted, Sutureless surgical procedures, Pain, postoperative, Wound healing, Edema

## Abstract

**Background:**

Although wisdom-tooth extraction is a routine intervention, the postoperative period remains marked by local inflammation classically manifesting as pain, edema and trismus. Furthermore, there is no consensus on the best operative techniques, particularly for the mucosal closure stage on impacted mandibular wisdom teeth.

**Methods:**

This parallel, randomized, non-blinded study compared pain following removal of impacted third molars, with and without sutures. Patients were electronically allocated 1:1 to extraction with versus without sutures. Patients ≥ 14 years’ old scheduled for extraction of four impacted wisdom teeth under general anesthesia at three French hospitals were eligible for inclusion. Exclusion criteria included taking antiplatelet agents or anticoagulants, coagulation disorders or immunosuppression, and planned orofacial surgical procedures or emergency pain/infection. The primary objective was pain evaluated by Visual Analogue Scale on Day 3. Secondary outcomes were edema, trismus, healing, complications, painkiller consumption and quality of life on Day 3 and 31.

**Results:**

Between June 2016 and November 2018, 100 patients were randomized. Finally, 44 patients in the Suture group and 50 patients in the Without Suture group were analyzed. Mean age was 16.5 years and 66.6% of patients were female. After adjustment on center, age and smoking, no statistical difference was seen between groups for pain on Day 3 (*p* = 0.904). No differences were seen for swelling, trismus, consumption of painkillers, healing, complications or quality of life. Smokers had a 3.65 times higher complications rate (*p* = 0.0244).

**Conclusions:**

Sutureless removal of third molars is thus a reliable technique without negative consequence on outcomes, and allows shorter operating time. Smoking is a risk factor for postoperative complications.

*Trial registration* www.clinicaltrials.gov (NCT02583997), registered 22/10/2015

## Background

Wisdom teeth extraction is a routine intervention and one of the most commonly performed in oral and maxillofacial surgery. The impaction rate of third molars is 24.40% [95% CI 18.79–30.82], with considerable differences according to geographical area [1]. These differences may be partially due to genetic factors, but are more likely to arise from environmental features [1]. The HAS (French Health Authority) states that closing the operating site is not compulsory, but advisable [2]. Despite consensus on the indications for removal [2], questions remain on the best operative techniques, especially the mucosal closure stage on impacted mandibular wisdom teeth. The method of healing by primary or secondary intention is particularly controversial. Some groups advocate for suturing sockets after extraction, with the rationale that wound approximation, limiting bleeding and decontaminating the postoperative site improve the quality and speed of healing. Yet others prefer secondary intention healing to naturally drain the operating site and thus reduce the risk of infection and inflammatory reaction, whilst maintaining site closure via cheek pressure. Dubois et al. [3] compared hermetic closure against sites where the mesial part of the wound was left to heal via secondary intention. By day 5, half of the hermetically sutured patients showed wound disunion, although without infection. Furthermore, although they found no significant difference in postoperative edema, pain or infection, there was a tendency for incomplete closure in sutured patients. Subsequent studies have compared operating site closure techniques following impacted wisdom tooth extraction [4–6]. However, none has evaluated pain, edema, trismus, complications, painkiller use and quality of life beyond 7 days postoperatively in a large cohort. A meta-analysis of five studies found reduced pain with secondary closure, although the results showed high heterogeneity due to the difference in incision techniques used [7]. Currently, there is insufficient evidence on whether primary or secondary healing is better for alveolar osteitis, infection or bleeding [7].

### Aims

Our primary objective was to compare postoperative pain at Day 3 in patients undergoing extraction of four impacted wisdom teeth, with or without sutures. The secondary objectives were to compare the operating time, long-term pain, edema and trismus, complications, correct flap repositioning, consumption of painkillers, the impact of smoking on complications, and quality of life.

## Methods

### Study design

This study was registered on clinicaltrials.gov (NCT02583997) and complies with the CONSORT guidelines. This was a non-blinded, randomized control trial with two parallel (1:1) arms. Patients were candidates for extraction of four impacted wisdom teeth under general anesthesia, aged ≥ 14 years old recruited from the university hospitals of Nîmes, Montpellier and Perpignan. Exclusion criteria were: pregnancy, breastfeeding or parturient, patients taking antiplatelet agents or anticoagulants, patients with coagulation disorders or immunosuppression, patients whose wisdom teeth were in a normal, functional, healthy position, if other orofacial surgical procedures were scheduled, and patients with emergency pain or infection. Four surgeons each in Perpignan and Montpellier and six surgeons in Nîmes were in charge of recruitment, randomization, surgery and follow-up.

Patient details (age, sex, weight, height, smoking habits) and surgical indication were recorded. The difficulty of tooth extraction (Winter classification) and root position regarding the inferior alveolar nerve (M3 to nerve proximity) were estimated. Baseline measurements were taken for pain, trismus and edema.

### Surgical procedure

Patients received oral premedication with 3 mg of bromazepam one hour before anesthesia and antibiotic prophylaxis with 2 g of amoxicillin. Patients underwent nasotracheal intubation. Intravenous anesthesia was induced with propofol and remifentanil via target-controlled infusion and maintained to keep the blood pressure and heartrate to within 20% of preoperative levels. Teeth were infiltrated with 2 ml of ropivacaine (7.5 mg/ml) prior to incision. For the lower jaw, a sulcular incision was made around the second mandibular molar with a retro-molar incision at the level of the ascending branch. Then we proceeded with mucoperiosteal detachment to the external oblique line, without detaching the papilla between the second premolar and the first molar. After osseous drilling, the crown was sectioned with cold irrigation, and fragments were extracted. The alveolus was curetted and the peri-coronary sac removed. The wound was washed with saline solution without alveolar dressing. Bone splinters were excised with Gouge forceps. In the Suture group, the lower jaw wounds were sutured with vicryl 4.0 using either single or two stitches, or a cross. In the Without Suture group, the flap was returned to its original position and maintained by jaw pressure. Hemostasis was checked. If necessary, patients in the Without Suture group could be given sutures in case of bleeding or inadequate flap repositioning. Intraoral compresses were inserted for 15 min. Ketamine was administered as a 0.3 mg/kg bolus at induction to prevent postoperative pain, and paracetamol, nefopam and ketoprofene were administered 20 min before the end of the intervention. Ondansetron (4 mg) and dexamethasone (4 mg) were given to prevent nausea. In the recovery ward, external freezing of the operated zone was offered to control pain. At discharge, patients were prescribed paracetamol and tramadol for 5 days and a mouthwash (chlorexidine) for 10 days. Patients had follow-up visits on Day 3 and 31 in which the wound site was evaluated and patients completed the Geriatric Oral Health Assessment Index (GOHAI) and gave their pain score on VAS. Analgesic use was assessed using a questionnaire given to patients at discharge to be completed daily and collected at the follow-up visit on Day 31.

### Outcome measures

The primary outcome was pain on Day 3 on a 0–10 Visual Analogic Scale (VAS). Pain on Day 0 and Day 31 was a secondary outcome. Remaining secondary outcomes were edema, trismus, complications, good flap healing in the experimental arm, and analgesic consumption on Days 0, 3 and 31, quality of life on Days 0 and 31, impact of smoking on complications, and operational time.

The operating time was calculated from first incision to the final removal of sterile drapes. Percentage edema was calculated as (D − D_baseline_)/D_baseline_)*100, where D = [(the distance from the left ear lobe to the left labial commissure) + (the distance from the right ear lobe to the right labial commissure)]/2. Trismus was measured as (T − T_baseline_)/T_baseline_)*100, where T = maximum mouth opening in mm. Flap healing was assessed (yes/no) for each tooth according to attachment loss at the second molar.

Complications recorded were: hemorrhage (continuous or intermittent bleeding from the socket immediately after extraction or later); infection (purulent discharge, a collection of pus or cellulitis); dry alveolitis (empty socket with a whitish, atonal bone giving off a foul odor and very sensitive); and suppurative alveolitis (presence of granulomatous tissue, bleeding and pus in the socket, accompanied by pain, trismus, low-grade fever, and regional lymphadenopathy). Quality of life was assessed by the 12-item GOHAI, assessing the frequency of problems in daily living due to dental situation with a final score ranging from 12 to 60 [8].

Blinding was not possible, however patients were not informed of their group and would have had difficulty inspecting their wounds in the short-term, thus patient blinding was considered likely for the first 3 days.

### Sample size calculation

Previous studies using similar techniques have shown a difference in pain on Day 2 of 0.7 ± 0.5 (size effect = 1.4)^3^, of 0.27 ± 0.715 on Day 7 (size effect = 0.38)^7^, and of 1 ± 1.07 (effect size = 0.93) on Day 3^8^. Anticipating a 10% loss to follow-up, a sample size of 100 patients was thus fixed to obtain 90 evaluable patients in order to highlight a size effect of at least 0.7 with a power of 90% and a 5% alpha risk.

### Data collection and analysis

A randomization list was created by the methodologist using SAS software (Cary, NC, USA) for each center using blocks of random size. Quantitative data were expressed as means and standard deviations or medians and interquartile ranges, according to their distribution. Qualitative data were expressed as absolute number and frequency (%). Comparison between groups used the Student T-test, Wilcoxon, chi-squared, or Fisher’s Exact tests as appropriate.

On Day 3, pain measured on the VAS was compared between the two groups via a generalized linear model to adjust the comparison on center, age and smoking. The analyses of secondary outcomes were adjusted on the center. Quantitative variables were analyzed in the same way as the primary outcome. Secondary outcomes, measured at several time points were compared between the two groups using mixed models with patient as random effect. The complication rate was compared between the two groups by a logistics regression model with adjustment on smoking and center. Patients deviating from the protocol were not replaced, as the study was conducted on an intention-to-treat basis. A *p*-value < 0.05 was considered as statistically significant. Statistical analysis was performed with R 3.5.1 software (R Development Core Team, (2018). R Foundation for Statistical Computing, Vienna, Austria).

## Results

### Participants

**Fig. 1:**
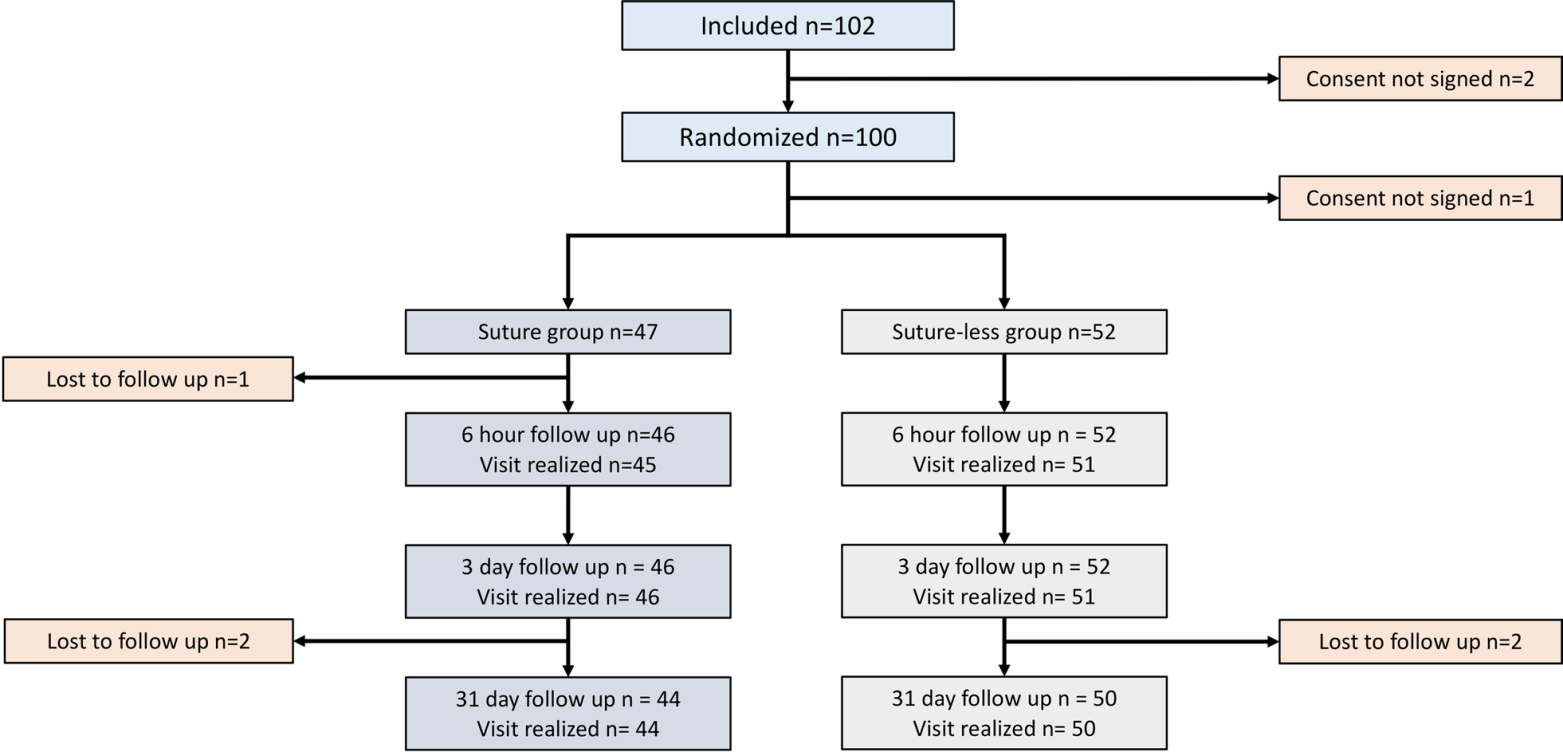
Flowchart

Between 02/06/2016 and 19/11/2018, 102 patients were recruited, with two excluded prior to randomization for non-signed consent forms, thus 48 patients were randomized to the Suture group and 52 to the Without Suture group. One patient in the Suture group did not sign the consent form, leaving 47 patients in this arm. Three patients in the With Suture group and two in the Without Suture groups were lost to follow-up (Fig. 1). Three patients were included despite not respecting the inclusion criteria: two with wisdom teeth in normal position in the Suture group, and one undergoing other orofacial surgical procedures in the Without Suture group. Four patients in the Without Suture group received sutures due to complications (intraoperative bleeding/hemorrhage (n = 2), mandible tooth bleeding 6 h postoperatively (n = 1) and mucosal spot at site 38–48 (n = 1)), although these patients were retained in the original group based on the intention-to-treat analysis. Only the affected tooth complication was sutured in these cases.

Patient characteristics are summarized in Table 1. Females were overrepresented in both groups: 59.6% patients in the Suture group and 73.1% in the Without Suture group. Smoking prevalence appeared to differ between groups, with 23.4% smokers in the Suture group versus 13.5% in the Without Suture group. The most common reason for the surgery was orthodontic, and the teeth were mainly mesial towards the front or vertical for both groups. For over 70% of cases, there was no superposition of the lower alveolar nerve with the dental roots. Most cases were not considered difficult, and these cases were equally split between groups.

**Table 1:** Patient baseline data

Inclusion characteristics	Missing	Suture (n = 47)	Missing	Without suture (n = 52)
Sex (female)	0	28 (59.6%)	0	38 (73.1%)
Age (years)	0	17 [15–19] (14–28)	0	16 [15–18] (14–27)
< 18 years’ old	0	31 (66%)	0	38 (73.1%)
Height	0	168.6 ± 9.0	0	166.5 ± 9.5
Weight	0	61.3 ± 11.4	0	58.4 ± 10.5
Body Mass Index	0	21.1 [19.4–22]	0	20.5 [19.3;22.3]
Smokers	0	11 (23.4%)	0	7 (13.5%)
Number of packets/year	3	1.5 [0.8;6.3]	1	7 [2.6;8.6]
Surgery indication				
Orthodontic	0	30 (63.8%)	0	42 (80.8%)
Pericoronitis	0	6 (12.8%)	0	0 (0%)
Pain, infection	0	3 (6.4%)	0	1 (1.9%)
Discomfort, pain alone	0	6 (12.8%)	0	2 (3.8%)
Lack of space	0	12 (25.5%)	0	13 (25%)
Distance lobe-commissure left (mm)	0	100 [95;104.5]	0	97.5 [94;103.25]
Distance lobe-commissure right (mm)	0	100 [95;105]	0	98 [93;104]
Trismus (mm)	1	45 [41; 50]	1	44 [40;48.5]
VAS pain	2	0 [0–0] (0–5)	1	0 [0–0] (0–8)
GOHAI total score	1	52.5 ± 6	1	53.8 ± 4.9
Winter classification				
Mesioangular	0		1	
Right lower		28 (59.6%)		32 (62.7%)
Left lower		30 (63.8%)		35 (68.6%)
Right upper		5 (10.6%)		4 (7.8%)
Left upper		6 (12.8%)		3 (5.9%)
Horizontal	0		1	
Right lower		2 (4.3%)		2 (3.9%)
Left lower		2 (4.3%)		0
Right upper		1 (2.1%)		0
Left upper		2 (4.3%)		0
Vertical	0		1	
Right lower		14 (29.8%)		15 (29.4%)
Left lower		13 (27.7%)		15 (29.4%)
Right upper		30 (63.8%)		33 (64.7%)
Left upper		27 (57.4%)		36 (70.6%)
Distoangular	0		1	
Right lower		1 (2.1%)		1 (2%)
Left lower		2 (4.3%)		1 (2%)
Right upper		10 (21.3%)		14 (27.5%)
Left upper		11 (23.4%)		12 (23.5%)
Transversal	0		1	
Right lower		2 (4.3%)		1 (2%)
Left lower		0		0
Right upper		1 (2.1%)		0
Left upper		1 (2.1%)		0
*Proximity of nerve to M3*				
No superposition	0		0	
Right lower		37 (78.7%)		38 (73.1%)
Left lower		34 (72.3%)		39 (75%)
Superposition	0		0	
Right lower		0		1 (1.9%)
Left lower		0		2 (3.8%)
Superposition without canal deformity	0		0	
Right lower		9 (19.1%)		11 (21.2%)
Left lower		11 (23.4%)		9 (17.3%)
Superposition with canal deformity	0		0	
Right lower			1 (2.1%)	2 (3.8%)
Left lower	0		2 (4.3%)	2 (3.8%)
Superposition with multiple deformities	0		0	
Right lower		0		0
Left lower		0		0

Data are presented as absolute number (%), mean ± standard deviation, median [IQR] (range)

Winter classification and proximity of nerve to M3 percentages have been calculated by area (right lower, left lower, right upper, left upper)

### Outcomes and estimation

Pain on Day 3 was low (2.72 ± 2.18 Suture vs. 2.54 ± 2.29 Without Suture, *p* = 0.68). After adjustment on center, age and smoking, no significant difference in pain was observed between the two arms (*p* = 0.904). The center effect (*p* = 0.26 for Montpellier versus Nîmes and *p* = 0.51 for Perpignan versus Nîmes) and age (*p* = 0.463) was also not significant. Smokers, however, had a 1.94-point higher VAS on Day 3 than non-smokers (*p* = 0.0084) (Table 2).

**Table 2:** Adjusted differences in outcome measures between arms

Pain D3 (VAS)	Estimate	Standard error	T value	*p*-value
Arm (Suture)	− 0.05	0.43	− 0.121	0.904
Center (Montpellier)	0.57	0.49	1.145	0.255
Center (Perpignan)	0.52	0.79	0.656	0.513
Age	0.06	0.08	0.737	0.463
Smoking	1.94	0.72	2.695	0.0084*
*Pain D31 (VAS)*				
Arm (Suture)	− 0.003	0.06	− 0.047	0.962
Center (Montpellier)	− 0.03	0.07	− 0.477	0.634
Center (Perpignan)	− 0.05	0.12	− 0.388	0.699
Age	− 0.01	0.01	− 1.057	0.294
Smoking	0.11	0.10	1.093	0.277
*Operating time (min)*				
Arm (Without Suture)	− 3.64	1.80	− 2.020	0.046*
Center (Montpellier)	17.38	2.05	8.478	< 0.001
Center (Perpignan)	− 6.26	3.36	− 1.865	0.065
*Edema D0 (mm)*				
Arm (Without Suture)	− 1.65	1.09	− 1.513	0.134
Center (Montpellier)	1.21	1.24	0.976	0.332
Center (Perpignan)	0.79	1.0	0.397	0.692
*Edema D3 (mm)*				
Arm (Without Suture)	− 1.42	1.69	− 0.842	0.402
Center (Montpellier)	1.37	1.93	0.712	0.478
Center (Perpignan)	− 0.69	3.10	− 0.223	0.824
*Edema D31 (mm)*				
Arm (Without Suture)	1.677	2.43	0.690	0.492
Center (Montpellier)	− 2.92	2.75	− 1.062	0.291
Center (Perpignan)	1.04	4.69	0.222	0.825
*GOHAI score D0*				
Arm (Without Suture)	1.12	1.07	1.039	0.301
Center (Montpellier)	0.021	1.24	0.017	0.987
Center (Perpignan)	5.14	1.98	2.598	0.011*
*GOHAI score D31*				
Arm (Without Suture)	0.89	1.76	0.506	0.614
Center (Montpellier)	3.74	1.99	1.877	0.064
Center (Perpignan)	7.49	3.39	2.210	0.030*
*GOHAI score D31*− *D3*				
Arm (Without Suture)	0.32	1.89	0.169	0.866
Center (Montpellier)	3.43	2.18	1.575	0.119
Center (Perpignan)	2.71	3.60	0.754	0.453
*Trismus D0 (mm)*				
Arm (Without Suture)	5.98	4.25	1.406	0.163
Center (Montpellier)	4.93	4.81	1.025	0.308
Center (Perpignan)	2.42	7.71	0.314	0.754
*Trismus D3 (mm)*				
Arm (Without Suture)	1.75	4.47	0.392	0.696
Center (Montpellier)	− 1.81	5.08	− 0.356	0.723
Center (Perpignan)	15.61	8.15	1.916	0.059
*Trismus D31 (mm)*				
Arm (Without Suture)	2.61	3.87	0.674	0.502
Center (Montpellier)	− 12.06	4.36	− 2.765	0.007*
Center (Perpignan)	2.44	7.41	0.329	0.743
*Complications*				
Arm (Suture)	0.69	0.50	1.374	0.170
Center (Montpellier)	0.41	0.54	0.748	0.454
Center (Perpignan)	− 0.35	1.13	− 0.309	0.758
Smoking	1.29	0.58	2.251	0.024*

* means *p* value ≤ 0.05

Data are adjusted according to center, age and smoking status

Patients generally did not report pain by Day 31 (median pain 0 [IQR 0; 0] Suture vs 0 [IQR 0; 0] Without Suture), with no difference between groups after adjustment (*p* = 0.962). The center effect (*p* = 0.63 for Montpellier versus Nîmes and *p* = 0.7 for Perpignan versus Nîmes), age (*p* = 0.294) and smoking status (*p* = 0.277) were also non-significant.

After adjustment on the center, operating time was 3.6 min shorter in the Without Suture group (*p* = 0.046) (Table 2). There was a center effect, with an operating time 17 min longer in the Montpellier center compared with Nîmes (*p* < 0.001) and 6.3 min shorter for Perpignan, without reaching significance (*p* = 0.065). There was no interaction between group and center. Edema was not significantly different between groups (*p* = 0.13 on Day 0, *p* = 0.40 on Day 2 and *p* = 0.49 on Day 31) after adjustment on the center. An analysis on repeated measures with a random effects model (patient effect) confirmed the absence of a significant difference between groups (*p* = 0.4038) after adjustment on the center, but we noted a significant decrease of 4.8% in edema between the immediately postoperative time point and Day 31 (*p* = 0.0085).

The variation in trismus was not significant between the two groups (*p* = 0.16 on Day 0, *p* = 0.70 on Day 3 and *p* = 0.50 on D31). However, it was 12% lower for Montpellier than Nîmes on Day 31 (*p* = 0.007) and 15% higher on Day 3 in Perpignan (*p* = 0.059). After a repeated measures analysis with a random effects model (patient effect), we confirmed no difference between groups (*p* = 0.1643), but a significant difference of − 18.8% in the variation relative to trismus was observed between the day of extraction and Day 3 (*p* < 0.001) and 31.2% between the day of extraction and Day 31 (*p* < 0.001), as well as a significant interaction between time (Day 31 versus day of extraction) and center (Montpellier vs. Nîmes) (*p* = 0.0033). No difference in complications rate was observed between the two groups (*p* = 0.14). However, the likelihood of onset of at least one complication during the study, adjusted on the center and group, was 3.65 times higher for smokers than non-smokers (*p* = 0.0244). There was a statistical trend for complications recorded for mandibular tooth infection with 17% in the Suture group versus 5.8% in the Without Suture (*p* = 0.075) on Day 31 (Table 3). No severe anesthetic reactions were recorded.

**Table 3:** Complications

D0 immediate postoperative	Suture (n = 47)	Without suture (n = 52)
Neighboring tooth or jaw fracture	1	0
Hemorrhage	1	0
Anesthesia reactions	0	1
*6 h postoperative*		
Mandibular tooth bleeding	3	1
*D3*^*a*^		
Maxillary tooth bleeding	1	1
Mandibular tooth bleeding	1	1
Mandibular tooth infection	2	0
Inflammatory responses	3	0
Nerve injury	1	1
Dry alveolitis	1	1
Suppurative alveolitis	1	0
*D31*^*b*^		
Maxillary tooth infection	0	1
Mandibular tooth infection	8	3
Inflammatory responses	4	1
Dry alveolitis	1	1
Suppurative alveolitis	1	1

^a^1 patient experienced 4 complications, 1 experienced 3, and 2 experienced 1,

^b^1 patient experienced 3 complications and 1 experienced 2

Painkiller consumption and local use of analgesics were not different between groups (Table 4) although a trend was seen for a higher rate of recourse to secondary analgesics in the Suture group (*p* = 0.057). Finally, quality of life was not affected by the suture, even after center adjustment (Table 2). However, global GOHAI score was statistically higher in Perpignan (5.1 points, *p* = 0.030), and higher though without reaching significance in Montpellier (3.1 points, *p* = 0.064) at D31, compared to Nîmes.

**Table 4:** Differences in analgesic use, healing and complications between groups

Painkiller consumption	Missing	Suture (n = 47)	Missing	Without suture (n = 52)	*p*-value
6 h postoperative	0	27 (57.4%)	0	35 (67.3%)	0.42
D3	4	43 (100%)	1	50 (98%)	1
D31	3	43 (97.7%)	1	48 (94.1%)	0.72
*Use of secondary analgesic tools*					
6 h postoperative	1	45 (97.8%)	1	49 (96.1%)	1
D3	4	43 (100%)	1	49 (96.1%)	0.55
D31	3	43 (97.7%)	2	42 (84%)	0.057
*Flap healing*					
6 h postoperative	1	46 (100%)	1	51 (100%)	1
D3	1	44 (95.7%)	1	49 (96.1%)	1
D31	3	44 (100%)	2	47 (94%)	0.29
*Complications*					
Immediate postoperative	0	2 (4.3%)	0	1 (1.9%)	0.93
6 h postoperative	1	3 (6.5%)	1	1 (2%)	0.53
D3	1	4 (8.7%)	1	3 (5.9%)	0.89
D31	3	9 (20.5%)	2	6 (12%)	0.40
At least one complication during the study	0	15 (31.9%)	0	9 (17.3%)	0.14

## Discussion

We found that both short- and long-term pain did not differ according to presence of sutures. Three similar studies have found better postoperative sequelae in non-sutured wounds. Osunde et al. [4] performed a randomized study on 83 patients with either multiple sutures or no sutures. Pain, edema and trismus were significantly higher in the Suture group up to Day 2, with no further difference up to Day 7. In our study, no significant difference was found up until Day 31. Another randomized controlled split-mouth study on 35 patients comparing a single stitch behind the second molar against no sutures found greater pain on the non-sutured side from Day 5 onwards, but this was non-significant before Day 5 [6]. Finally, Mahat et al. [5] performed a randomized study of 48 patients with either hermetic sutures with separate stitches or without suture, showing that pain was statistically higher for the Suture group only on Day 1.

Smokers showed 3.65 times more complications than non-smokers. A systematic review suggested tobacco induced dry sockets, especially in the first 24 h [9], possibly due to the sucking motion during smoking dislodging the clot [10], or smoking leading to granulation tissues and a decreased local immune and inflammatory response [11]. The postoperative antibiotics given in the Mahat et al. study, alongside thorough postoperative instructions, may have avoided dry sockets. Unfortunately, Osunde et al. excluded smokers from their study [4].

We found no between-group differences for trismus, painkiller consumption, postoperative complications and edema, similarly to Mahat et al. [5]. In contrast, Alkadi et al. observed significantly better healing up to one month on the sutured side, but without difference in edema and bleeding between the two techniques, evaluated up to Day 7 [6]. However, edema was significantly higher in the Suture group in the Mahat et al. study [5] when measured between the mandibular angle and the lateral cantus, whilst edema measured between the tragus and the labial commissure was not significantly different. Osunde et al. presented edema as the mean of the two measurements and found statistically less edema in the no-suture group until Day 2, but no difference between Day 3 and 7 [4]. Alkadi et al. recorded edema on a six-point scale without statistical differences until Day 7 between the sutured and non-sutured sides [6].

We found no difference between groups in quality of life. Considering postoperative quality of life relative to limitation of daily activities, measured as return to work the day after surgery, Mahat et al. only found this limitation in the Suture group [5]. Excessive pain or social interaction limitation could likely be extrapolated as inability to work, but could vary between people.

The heterogeneity of the studies means they should be compared with caution. Our study was unique in offering anxiolytic premedication and general anesthesia; the others used local anesthesia with or without intravenous sedation [4, 5]. A major strength of our study was the multicentric design, with several operators and evaluators, whereas other studies used a single surgeon who also performed the outcome evaluation [4, 5]), or a single operator and several evaluators [6]. We only gave prophylactic antibiotic treatment, whereas Osunde et al. [4] and Mahat et al. [5] applied a postoperative antibiotic therapy and Alkadi et al. [6] gave both prophylactic antibiotic treatment and antibiotic therapy. Mahat et al. [5] and Osunde et al. [4] made mesial relief incisions combined with a multiple-stitch silk thread suture, requiring subsequent removal. In contrast, Alkadi et al. opted for an incision without mesial relief and a single-stitch vicryl resorbable suture [6], as we did.

The number of teeth requiring removal and extent of impaction also differed between studies. These differences can lead to bias in comparison with these studies, particularly with Mahat et al. [5] due to the difference between totally and partially impacted third molar as a starting point concerning the difficulty of surgery or severity of the final wound.

This study had several limitations. Despite randomization, sex ratio and smoking prevalence appeared different between groups, both of which could potentially alter the results. However, adjustment on smoking status was planned in the protocol. Hence, the potential bias of smoking was accounted for in the results. Patients completed the questionnaires on Day 31, retrospectively recording the period starting from Day 2, which could explain the high scores even after several weeks. The practitioner was informed of the randomization arm at the beginning of surgery. Waiting until the end of the intervention to reveal the group could have avoided a bias. We did not note the type of suture used, however, certain studies have shown differences between different types of suture [4–6].

Questions remain over the best surgical techniques to use during extraction. A meta-analysis failed to find a superior technique on postoperative sequelae using different shaped access flaps [12]. In contrast, a meta-analysis of mucous closure techniques highlighted a significantly favorable effect on edema of a closure preceded by exeresis of a gingival flap, disto-vestibular to the second molar compared with a classical hermetic mucous closure [13]. Gay-Escoda et al. [14] found no significant postoperative differences between a mesial slot incision, sutured hermetically or not.

Surgical drainage presents an interesting avenue for further study, but is little used, with no real agreement as regards pain, edema or trismus [15]. A 2012 systematic review comparing hermetic suture techniques with various closure protocols favoring secondary healing (drainage, gauze strip, single-stich suture and exeresis of a mucous flap) could not confirm the superiority of one technique over another for impact of edema, trismus, postoperative complications and pain [16]. A Cochrane review found that antibiotic prophylaxis decreased the risk of infections, with a RR of 0.34 [95% CI 0.19–0.64], and also reduced occurrence of dry socket [17]. However, the results were inconclusive on the effect on pain. Nevertheless, this reduced risk needs to be balanced against the advice to limit antibiotics to avoid resistance [17].

Finally, numerous studies have broached the question of adjuvant surgery to improve sequelae. Brković et al. [18] found that ropivacaine as supplemental injection provides a longer duration of postoperative analgesia, compared to placebo. Sub-mucosal dexamethasone injection had a significant beneficial postoperative effect on pain and trismus on the operating site [19], although there was no difference in late pain between the sub-mucosal, intravenous or intramuscular routes of administration [20]. Installation of a collagen sponge in the alveoli statistically significantly reduced pain [21, 22]. A recent randomized controlled study found that ibuprofen and nimesulide produced better pain scores compared with acetaminophen, ketoprofen and dexamethasone when given as preemptive analgesia [23].

A meta-analysis studying the contribution of PRF (Platelet-rich Fibrin) in the sockets confirmed significant improvement regarding pain, edema and onset of osteitis [24]. A study demonstrated greater efficacy of irrigating the site with chlorhexidine versus saline solution and iodinated povidone on reducing postoperative pain, edema and trismus [25]. Reyes-Gilabert et al. [26] identified a statistically significant association between pre- and postoperative anxiety and also between postoperative anxiety and pain in oral surgery. Therefore preoperative per os sedation could be beneficial. In our case, patients had been given preoperative bromazepam.

## Conclusions

This study confirms the absence of significant differences of various outcomes following extraction of four impacted wisdom teeth, with and without suture. The sutureless technique reduces operating times and saves money. Adjustments were performed to remove bias such as smoking. Smoking was a statistically significant factor of the onset of postoperative complications. For impacted teeth with correct flap repositioning and without intraoperative hemorrhage, the sutureless operative technique does not require specific material or experience. As this technique does not present any difference in postoperative effects from a sutured technique, it should be used preferentially. Nevertheless, prospective works are now needed to assess the economic impact of the two techniques with and without sutures and to further evaluate the impact of smoking, as it can limit the generalizability of our study. The operative techniques that minimize complications for smokers may also be interesting to study.

## Data Availability

The full trial protocol and study data can be accessed on reasonable request to takadoumsarah@gmail.com or to christophe.demattei@chu-nimes.fr.

## Abbreviations

GOHAI: Geriatric oral health assessment index
HAS: Haute Autorité de Santé (French Health Authority)
VAS: Visual analog scale
